# Adipose-Derived Mesenchymal Stem Cell Protects Kidneys against Ischemia-Reperfusion Injury through Suppressing Oxidative Stress and Inflammatory Reaction

**DOI:** 10.1186/1479-5876-9-51

**Published:** 2011-05-05

**Authors:** Yen-Ta Chen, Cheuk-Kwan Sun, Yu-Chun Lin, Li-Teh Chang, Yung-Lung Chen, Tzu-Hsien Tsai, Sheng-Ying Chung, Sarah Chua, Ying-Hsien Kao, Chia-Hong Yen, Pei-Lin Shao, Kuan-Cheng Chang, Steve Leu, Hon-Kan Yip

**Affiliations:** 1 Division of Urology, Department of Surgery, Chang Gung Memorial Hospital - Kaohsiung Chang Gung Memorial Hospital and Chang Gung University College of Medicine, Kaohsiung, Taiwan; 2 Department of Emergency Medicine, E-Da Hospital, I-Shou University, Kaohsiung, Taiwan; 3 Division of Cardiology, Department of Internal Medicine, Chang Gung Memorial Hospital - Kaohsiung Chang Gung Memorial Hospital and Chang Gung University College of Medicine, Kaohsiung, Taiwan; 4 Center for Translational Research in Biomedical Sciences, Kaohsiung Chang Gung Memorial Hospital and Chang Gung University College of Medicine, Kaohsiung, Taiwan; 5 Basic Science, Nursing Department, Meiho University, Pingtung, Taiwan; 6 Department of Medical Research, E-DA Hospital, I-Shou University, Kaohsiung, Taiwan; 7 Department of Life Science, National Pingtung University of Science and Technology, Pingtung, Taiwan; 8 Graduate Institute of Medicine, College of Medicine, Kaohsiung Medical University, Kaohsiung, Taiwan; 9 Department of Cardiology, School of Medicine, China Medical University, Taichung, Taiwan; 10 Division of General Surgery, Department of Surgery, Chang Gung Memorial Hospital - Kaohsiung Chang Gung Memorial Hospital and Chang Gung University College of Medicine, Kaohsiung, Taiwan

## Abstract

**Background:**

Reactive oxygen species are important mediators exerting toxic effects on various organs during ischemia-reperfusion (IR) injury. We hypothesized that adipose-derived mesenchymal stem cells (ADMSCs) protect the kidney against oxidative stress and inflammatory stimuli in rat during renal IR injury.

**Methods:**

Adult male Sprague-Dawley (SD) rats (n = 24) were equally randomized into group 1 (sham control), group 2 (IR plus culture medium only), and group 3 (IR plus immediate intra-renal administration of 1.0 × 10^6 ^autologous ADMSCs, followed by intravenous ADMSCs at 6 h and 24 h after IR). The duration of ischemia was 1 h, followed by 72 hours of reperfusion before the animals were sacrificed.

**Results:**

Serum creatinine and blood urea nitrogen levels and the degree of histological abnormalities were markedly lower in group 3 than in group 2 (all p < 0.03). The mRNA expressions of inflammatory, oxidative stress, and apoptotic biomarkers were lower, whereas the anti-inflammatory, anti-oxidative, and anti-apoptotic biomarkers were higher in group 3 than in group 2 (all p < 0.03). Immunofluorescent staining showed a higher number of CD31+, von Willebrand Factor+, and heme oxygenase (HO)-1+ cells in group 3 than in group 2 (all p < 0.05). Western blot showed notably higher NAD(P)H quinone oxidoreductase 1 and HO-1 activities, two indicators of anti-oxidative capacity, in group 3 than those in group 2 (all p < 0.04). Immunohistochemical staining showed higher glutathione peroxidase and glutathione reductase activities in group 3 than in group 2 (all p < 0.02)

**Conclusion:**

ADMSC therapy minimized kidney damage after IR injury through suppressing oxidative stress and inflammatory response.

## Background

**N**ot only is ischemia-reperfusion (IR) injury of the kidney encountered in patients with contrast media-induced nephropathy [1] and in those with shock followed by resuscitation in the emergency and intensive care settings [2], but it is also a common early event in kidney transplantation that contributes to organ dysfunction [3]. The manifestations include acute tubular-epithelial damage [4,5], loss of peri-tubular microvasculature [6], as well as inflammation and leukocyte infiltration [3-5,7]. Despite current advances in medical treatment, IR injury of the kidney, which is a common cause of acute renal failure, remains a major healthcare problem with high rates of in-hospital mortality and morbidity [4,8,9]. This situation warrants the development of new treatment modalities [7].

Growing data have shed considerable light on the effectiveness and safety of mesenchymal stem cell (MSC) treatment in improving ischemia-related organ dysfunction [7,10-12]. Indeed, the therapeutic potential of MSC has been extensively investigated using animal models of kidney disease [7,10,11,13]. Interestingly, although several experimental studies [6,7,10,11,13-15] have established the role of MSC therapy in preserving renal parenchymal integrity from acute ischemic injury and improving kidney function from acute damage through engraftment of MSCs in both glomerular and tubular structures, regeneration of tubular epithelium, augmentation of paracrine and systemic secretory functions, and enhancement of peri-tubular capillary regeneration, the precise mechanisms underlying the improvement in kidney function remain unclear. Furthermore, despite the availability of various cellular sources for experimental investigations [6,7,10-15] including bone marrow-derived mesenchymal stem cells (BMDMSCs), hematopoietic stem/progenitor cells, and cells of embryonic origins, the ethical issue regarding the source and safety of allo- and xeno-grafting has become important concern in the clinical setting. On the other hand, the use of adipose-derived (AD) MSCs has the distinct advantages of minimal invasiveness in harvesting and unlimited supply from in vitro culturing [16]. In addition, the paracrine characteristics of ADMSCs have been shown to be different from those of bone marrow origin with the former showing more potent anti-inflammatory and immuno-modulating functions [17]. Moreover, although it has been reported that the complicated mechanisms underlying IR injuries of solid organs involve the generation of reactive oxygen species (ROS), mitochondrial damage [18,19], apoptosis [7], and a cascade of inflammatory processes [6], the impact of MSCs treatment on these cellular and molecular changes [6,7,18,19] during renal IR injury remains to be elucidated. Therefore, we hypothesized that administration of ADMSCs is beneficial in alleviating IR injury of the kidney through ameliorating anti-inflammatory response and oxidative stress as well as preserving the integrity of peri-tubular microvasculature.

## Methods

### Ethics

All experimental animal procedures were approved by the Institute of Animal Care and Use Committee at our hospital and performed in accordance with the Guide for the Care and Use of Laboratory Animals (NIH publication No. 85-23, National Academy Press, Washington, DC, USA, revised 1996).

### Animal Grouping and Isolation of Adipose-Derived Mesenchymal Stem Cells

Pathogen-free, adult male Sprague-Dawley (SD) rats (n = 24) weighing 275-300 g (Charles River Technology, BioLASCO Taiwan Co., Ltd., Taiwan) were randomized into group 1 (sham control), group 2 (IR plus culture medium) and group 3 (IR plus autologous ADMSC implantation) before isolation of ADMSCs.

The rats in group 3 (n = 8) were anesthetized with inhalational isoflurane 14 days before induction of IR injury. Adipose tissue surrounding the epididymis was carefully dissected and excised. Then 200-300 μL of sterile saline was added to every 0.5 g of tissue to prevent dehydration. The tissue was cut into <1 mm^3 ^size pieces using a pair of sharp, sterile surgical scissors. Sterile saline (37°C) was added to the homogenized adipose tissue in a ratio of 3:1 (saline: adipose tissue), followed by the addition of stock collagenase solution to a final concentration of 0.5 units/mL. The centrifuge tubes with the contents were placed and secured on a Thermaline shaker and incubated with constant agitation for 60 ± 15 minutes at 37°C. After 40 minutes of incubation, the content was triturated with a 25 mL pipette for 2-3 minutes. The cells obtained were placed back to the rocker for incubation. The contents of the flask were transferred to 50 mL tubes after digestion, followed by centrifugation at 600 g for 5 minutes at room temperature. The lipid layer and saline supernatant from the tube were poured out gently in one smooth motion or removed using vacuum suction. The cell pellet thus obtained was resuspended in 40 mL saline and then centrifuged again at 600 g for 5 minutes at room temperature. After being resuspended again in 5 mL saline, the cell suspension was filtered through a 100 mm filter into a 50 mL conical tube to which 2 mL of saline was added to rinse the remaining cells through the filter. The flow-through was pipetted into a new 50 mL conical tube through a 40 mm filter. The tubes were centrifuged for a third time at 600 g for 5 minutes at room temperature. The cells were resuspended in saline. An aliquot of cell suspension was then removed for cell culture in DMEM-low glucose medium containing 10% FBS for 14 days. Approximately 5.5 × 10^6 ^ADMSCs were obtained from each rat.

### Flow Cytometric Characterization of ADMSCs

**Figure 1:**
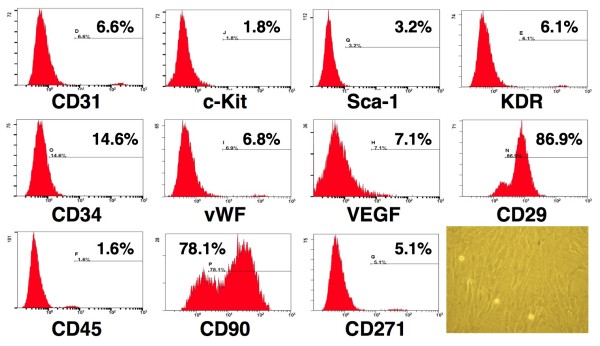
**Flow cytometric analysis of rat adipose-derived mesenchymal stem cells (ADMSCs)**. After culturing for 14 days, majority of isolated adipose-derived stem cells expressing CD29 and CD90 characteristic of mesenchymal stem cells (n = 3). Note the spindle-shaped morphological feature of the stem cells (Right lower panel) (200×).

Flow cytometric analysis was performed for identification of cellular characteristics after cell-labeling with appropriate antibodies 30 minutes before transplantation (Figure 1). Briefly, the cultured ADMSCs were washed twice with phosphate buffer solution (PBS) and centrifuged before incubation with 1 mL blocking buffer for 30 minutes at 4°C. After being washed twice with PBS, the cells were incubated for 30 minutes at 4°C in a dark room with the fluorescein isothiocyanate (FITC)-conjugated antibodies against CD34 (BD pharmingen), C-kit (BD pharmingen), Sca-1 (BD pharmingen), vWF (BioLeogend), VEGF (BD pharmingen) or the phycoerythrin (PE)-conjugated antibodies against CD31 (AbD serotec), kinase insert domain-conjugating receptor (KDR) (BD pharmingen), CD29 (BD pharmingen), CD45 (BD Bioscience), CD90 (BD Bioscience), CD 271 (BD pharmingen). Isotype-identical antibodies (IgG) served as controls. After staining, the cells were fixed with 1% paraformaldehyde. Flow cytometric analyses were performed by utilizing a fluorescence-activated cell sorter (Beckman Coulter FC500 flow cytometer). Cell viability of >95.0% was noted in each group. Assessment in each sample was performed in duplicate, with the mean level reported. LMD files were exported and analyzed using the CXP software.

### ADMSC Labeling, Protocol of IR Induction, and Rationale of Timing for ADMSC Administration

On day 14, CM-Dil (Vybrant™ Dil cell-labeling solution, Molecular Probes, Inc.) (50 μg/ml) was added to the culture medium 30 minutes before IR procedure for ADMSC labeling. The procedures of CM-Dil staining for ADMSC were performed based on our previous study [20]. After completion of ADMSC labeling, all animals were anesthetized by chloral hydrate (35 mg/kg i.p.) plus inhalational isoflurane and placed in a supine position on a warming pad at 37°C. Renal IR was then conducted in group 2 and group 3 animals on which a midline laparotomy was performed. Bilateral renal pedicles were clamped for one hour using non-traumatic vascular clips before reperfusion for 72 hours. Normal controls without renal IR (i.e. group 1) were subjected to laparotomy only.

Previous experimental study [21] has demonstrated that administration of mesenchymal stem cells either immediately or 24 h after IR-induced acute renal failure has significantly improved renal function and alleviated renal injury. In addition, we have recently shown that administration of ADMSCs to the rats at 6-hour intervals after acute ischemic stroke significantly improved organ damage [16]. Thus, the timing for ADMSC administration in the current study was based on these studies.

In group 2 animals, intra-renal injection of 35 μL of culture medium was performed one hour after reperfusion, followed by intra-venous injection of 35 μL culture medium at 6 and 24 hours after IR procedure through the penile vein. Group 3 animals followed the same protocol, except for that equal volume of culture medium with ADMSCs (1.0 × 10^6^) was administered at each time point instead of pure culture medium as in group 2. For the study purpose, animals were sacrificed at day 1 (n = 6), day 3 (n = 8) and day 14 (n = 6) after IR procedure. The kidneys were collected for subsequent studies.

### Determination of Renal Function

Serum levels of creatinine and blood urea nitrogen (BUN) were measured in all three groups of rats prior to IR procedure and at 24 h, 72 h, and day 14 after the IR procedure before sacrificing the animals (n = 6 for each group). Additionally, urine protein and creatinine levels were also measured in all animals at these time points. Twenty-four hour urine was collected from the study animals for estimating daily urine volume and measuring the ratio of urine protein to urine creatinine excretion. Quantification of urine protein, BUN, and creatinine level was performed using standard laboratory equipment at our hospital.

### Hematoxylin and Eosin (H & E) Staining and Histopathology Scoring

Kidney specimens from all animals were fixed in 10% buffered formalin before embedding in paraffin. Tissue was sectioned at 5 mm and then stained with hematoxylin and eosin for light microscopic analysis. Histopathology scoring was applied based on a previous study [22] in a blind fashion. The score was given based on grading of tubular necrosis, loss of brush border, cast formation, and tubular dilatation in 10 randomly chosen, non-overlapping fields (200×) as follows: 0 (none), 1 (≤10%), 2 (11-25%), 3 (26-45%), 4 (46-75%), and 5 (≥76%).

### Immunofluorescent and Immunohistochemical (IHC) Studies

CM-Dil-positive ADMSCs engrafted in the renal parenchyma after transplantation were identified through immunofluorescent staining that was also used for the examination of heme oxygenase (HO)-1-, CD31-, or vWF-positive cells using respective primary antibody. Moreover, IHC labeling technique was adopted for identifying glutathione peroxidase (GPx)- and glutathione reductase (GR)-positive cells using respective primary antibodies based on our recent study [20]. Irrelevant antibodies were used as controls in the current study.

An IHC-based scoring system was utilized for semiquantitative analyses of GR and GPx as percentage of positive cells in a blind fashion [Score of positively-stained cell for GR and GPx: 0 = no stain %; 1 = <15%; 2 = 15-25%; 3 = 25-50%; 4 = 50-75%; 5 = >75-100% per high-power filed (200 ×)].

### Western Blot Analysis for Oxidative Stress, Nuclear Factor (NF)-κB, Intercellular Adhesion Molecule (ICAM)-1, HO-1, NAD(P)H Quinone Oxidoreductase (NQO)1 in Kidney

Equal amounts (10-30 mg) of protein extracts from kidney were loaded and separated by SDS-PAGE using 8-10% acrylamide gradients. Following electrophoresis, the separated proteins were transferred electrophoretically to a polyvinylidene difluoride (PVDF) membrane (Amersham Biosciences). Nonspecific proteins were blocked by incubating the membrane in blocking buffer (5% nonfat dry milk in T-TBS containing 0.05% Tween 20) overnight. The membranes were incubated with the indicated primary antibodies (GR, 1: 1000, Abcam; NQO1, 1: 1000, Abcam; GPx, 1: 2000, Abcam; HO-1, 1: 250, Abcam; ICAM-1, 1: 2000, Abcam; NF-κB [p65], 1: 200, Santa Cruz; Actin 1: 10000, Chemicon) for 1 hour at room temperature. Horseradish peroxidase-conjugated anti-rabbit immunoglobulin IgG (1: 2000, Cell signaling) was used as a second antibody for 1 hour at room temperature. The washing procedure was repeated eight times within one hour.

The Oxyblot Oxidized Protein Detection Kit was purchased from Chemicon (S7150). The procedure of 2,4-dinitrophenylhydrazine (DNPH) derivatization was carried out on 6 μg of protein for 15 minutes according to manufacturer's instructions. One-dimensional electrophoresis was performed on 12% SDS/polyacrylamide gel after DNPH derivatization. Proteins were transferred to nitrocellulose membranes which were then incubated in the primary antibody solution (anti-DNP 1: 150) for two hours, followed by incubation with secondary antibody solution (1:300) for one hour at room temperature. The washing procedure was repeated eight times within 40 minutes.

Immunoreactive bands were visualized by enhanced chemiluminescence (ECL; Amersham Biosciences) which was then exposed to Biomax L film (Kodak). For quantification, ECL signals were digitized using Labwork software (UVP). For oxyblot protein analysis, a standard control was loaded on each gel.

### Real-Time Quantitative PCR Analysis

Real-time polymerase chain reaction (PCR) was conducted using LightCycler TaqMan Master (Roche, Germany) in a single capillary tube according to the manufacturer's guidelines for individual component concentrations as we previously reported [12,20]. Forward and reverse primers were each designed based on individual exons of the target gene sequence to avoid amplifying genomic DNA.

During PCR, the probe was hybridized to its complementary single-strand DNA sequence within the PCR target. As amplification occurred, the probe was degraded due to the exonuclease activity of Taq DNA polymerase, thereby separating the quencher from reporter dye during extension. During the entire amplification cycle, light emission increased exponentially. A positive result was determined by identifying the threshold cycle value at which reporter dye emission appeared above background.

### Statistical Analysis

Quantitative data are expressed as means ± SD. Statistical analysis was adequately performed by ANOVA followed by Bonferroni multiple-comparison post hoc test. Statistical analysis was performed using SAS statistical software for Windows version 8.2 (SAS institute, Cary, NC). A probability value <0.05 was considered statistically significant.

## Results

### Serial Changes in Serum Levels of Creatinine and BUN, Urine Amount and the Ratio of Urine Protein to Creatinine after IR Procedure

**Figure 2:**
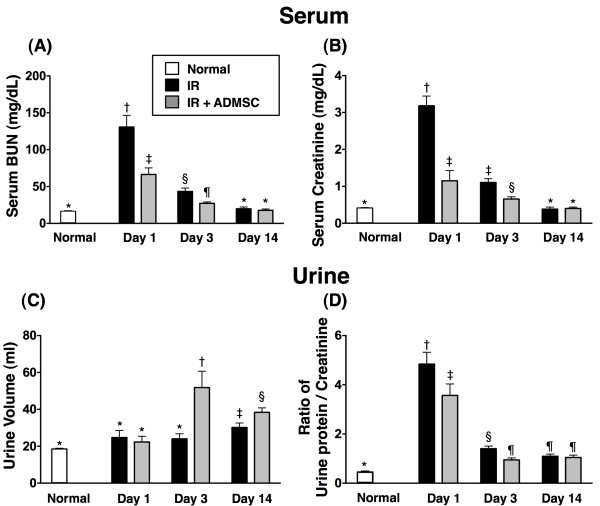
**Serial changes in serum levels of blood urea nitrogen (BUN) and creatinine, urine amount, and the ratio of urine protein to creatinine**. Serum levels of blood urea nitrogen (BUN) and creatinine and the ratio of urine protein to creatinine in three groups [control group; ischemia reperfusion (IR) group; IR + adipose-derived mesenchymal stem cell (ADMSC)] of rats on days 1, 3, and 14 after IR. **A) **For BUN: **1) **normal vs. day 1, *p < 0.0001 between the indicated groups; **2) **normal vs. day 3, *p < 0.02 between the indicated groups; **3) **normal vs. day 14, p > 0.5 between the indicated groups. **B) **For creatinine: **1) **normal vs. day 1, *p < 0.0001 between the indicated groups; **2) **normal vs. day 3, *p < 0.02 between the indicated groups; **3) **normal vs. day 14, *p > 0.5 between the indicated groups. **C) **Daily urine amount in thee groups of rats on days 1, 3, and 14 after IR injury. **1) **normal vs. day 1, *p > 0.5 between the indicated groups; **2) **normal vs. day 3, *p < 0.0001 between the indicated groups; **3) **normal vs. day 14, *p < 0.02 between the indicated groups. **D) **The ratio of urine protein to urine creatinine in three groups of rats on days 1, 3, and 14 after IR. **1) **normal vs. day 1, *p < 0.0001 between the indicated groups; **2) **normal vs. day 3, *p < 0.001 between the indicated groups; **3) **normal vs. day 14, *p < 0.02 between the indicated groups. Symbols (*, †, ‡, §, ¶) indicate significance (at 0.05 level) (by Bonferroni multiple comparison post hoc test).

Three time points (i.e. 24 h, 72 h, and day 14 after the IR procedure) were chosen for determining the serial changes in serum levels of creatinine and BUN (Figure 2A &2B). Both BUN and creatinine were notably higher in IR group (group 2) than those in normal controls (group 1) and the IR + ADMSC group (group 3), and remarkably higher in group 3 than in group 1 at 24 h and 72 h after IR procedure. However, these parameters did not differ among groups 1, 2, and 3 at day 14 after IR procedure. These findings indicate successful induction of renal IR injury in an experimental setting and significant attenuation of IR-elicited deterioration in renal function at acute phase after IR. In addition, the renal function recovered by day 14 after acute phase of IR injury.

The daily urine amount did not differ among groups 1, 2, and 3 at 24 h after IR procedure (Figure 2C). However, the amounts were remarkably increased in group 3 as compared with group 2 at 72 h and day 14 after IR procedure. Conversely, the ratio of urine protein to urine creatinine was notably lower in group 3 than in group 2 at 24 h and 72 h after IR (Figure 2D). However, the ratios were similar between groups 2 and 3 at day 14 after IR procedure.

### Histopathological Scoring of the Kidneys

**Figure 3:**
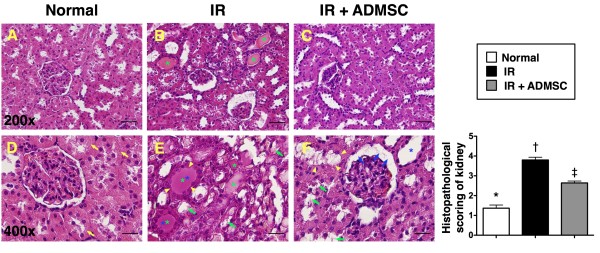
**Histopathological scoring of ischemia-reperfusion (IR)-induced renal injury**. H & E staining (200 × in A, B & C and 400 × in D, E & F) of kidney sections in normal, IR, and IR + ADMSC animals, showing notably higher degree of loss of brush border in renal tubules (yellow arrowheads), cast formation (green asterisk), tubular dilatation (blue asterisk), and tubular necrosis (green arrows) in IR without treatment group than in other groups. Also note dilatation of Bowman's capsule (blue arrows) in animals after IR with ADMSC treatment. *p < 0.03 between the indicated groups. Symbols (*, †) indicate significance (at 0.05 level) (by Bonferroni multiple comparison post hoc test). Scale bars in right lower corners represent 50 μm in A, B, & C, and 25 μm in D, E, & F.

To evaluate the impact of ADMSC transplantation on the severity of IR-induced renal injury, histological scoring based on the typical microscopic features of acute tubular damage, including extensive tubular necrosis and dilatation, as well as cast formation and loss of brush border was adopted (Figure 3). The injury was found to be more severe in group 2 than in group 3, suggesting that ADMSC therapy significantly protected the kidney from IR damage.

### Identification of ADMSC Engrafted into Renal Parenchyma and CD31+ and von Willebrand Factor (vWF)+ Cells in Peri-tubular Regions

**Figure 4:**
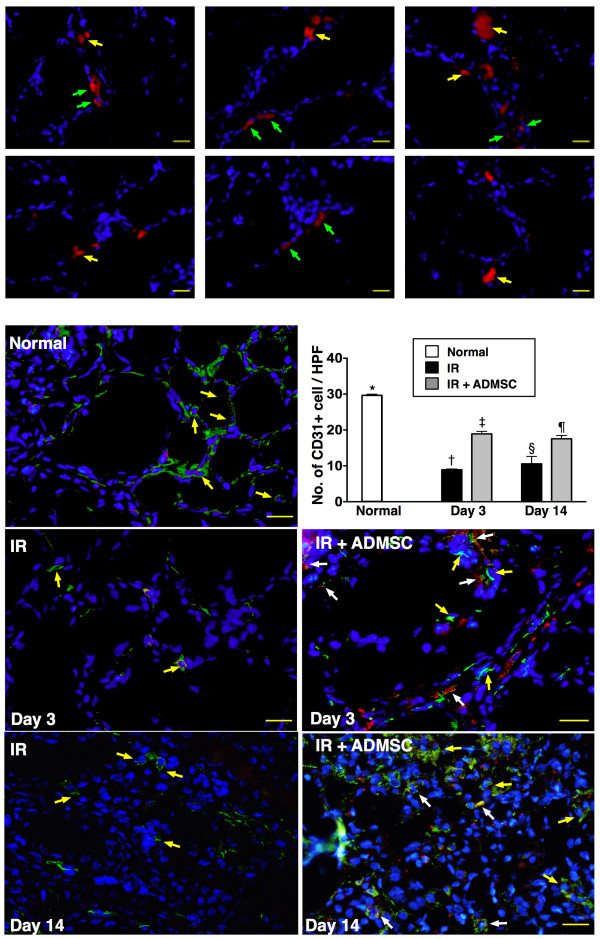
**Engraftment of adipose-derived mesenchymal stem cells (ADMSCs) in renal tissue after ischemia-reperfusion (IR) injury**. **Upper panel**: Identification of Dil-positive ADMSCs (red) (400 ×) in peri-tubular area (green arrows) and interstitial area of kidney (yellow arrows) 72 h post-IR. DAPI counter-staining for nuclei (blue). Scale bars at right lower corners represent 20 μm **Lower panel**: By days 3 and 14, notably higher number of CD31+ cells (yellow arrows) in control group than in IR and IR + ADMSC groups. Significantly increased number in IR + ADMSC group than in IR group (n = 8 in each group). Merged image from double staining with Dil + CD31 shown in "IR + ADMSC". Note numerous doubly-stained cells in peri-tubular and interstitial areas (white arrows). Scale bars at right lower corners represent 20 μm. **1) **Normal vs. day 3, *p < 0.001 between the indicated groups. **2) **Normal vs. day 14, *p < 0.001 between the indicated groups. Symbols (*, †, ‡, §, ¶) indicate significance (at 0.05 level) (by Bonferroni multiple comparison post hoc test).

**Figure 5:**
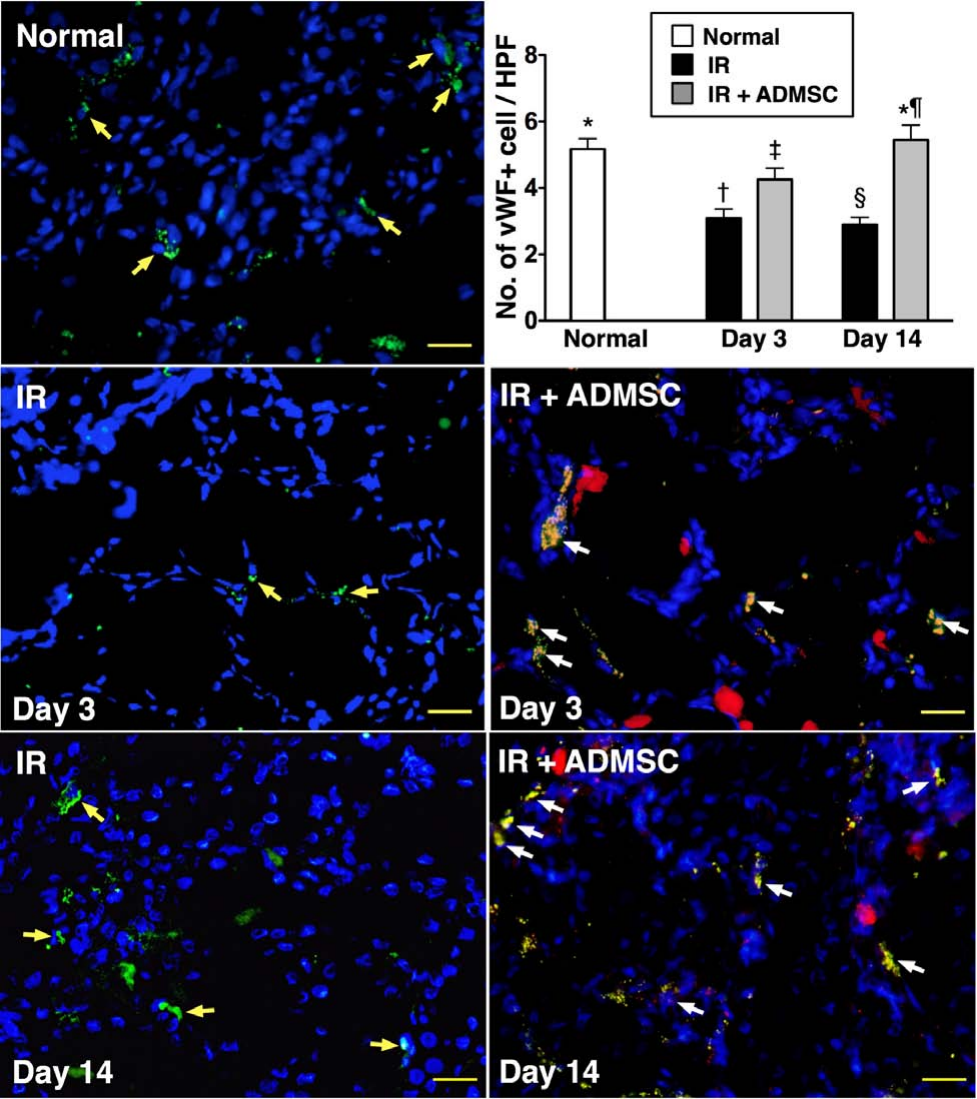
**Immunofluorescent staining of von Willebrand factor (vWF)-positive cells in peri-tubular and interstitial areas of kidney**. On day 3 after IR injury, notably higher number of vWF+ cells (yellow arrows) in control group than in IR and IR + ADMSC groups (400 ×), and significantly higher number in IR + ADMSC group than in IR group. By day 14 after IR procedure, markedly higher number of vWF+ cells (yellow arrows) in control group and IR + ADMSC groups than in IR group (400 ×), but no significant difference between control group and IR + ADMSC group. Merged image from double staining with Dil + vWF shown in "IR + ADMSC". Identification of numerous doubly-stained cells in peri-tubular and interstitial areas (white arrows). Scale bars at right lower corners represent 20 μm; **1) **normal vs. day 3, *p < 0.03 between the indicated groups; **2) **normal vs. day 14, *p < 0.03 between the indicated groups (n = 8 for each group). Symbols (*, †, ‡, §, ¶) indicate significance (at 0.05 level) (by Bonferroni multiple comparison post hoc test).

Under fluorescence microscope (Figure 4, upper panel), numerous CM-Dil-positive ADMSCs were identified in renal parenchyma of group 3 animals. Interestingly, most of these ADMSCs were found to engraft into interstitial and peri-tubular areas of kidney on day 3 after IR injury. Moreover, some cells positive for CD31 (Figure 4, lower panel) and vWF (Figure 5), indicators of endothelial phenotypes, were found to be located in interstitial and peri-tubular regions and some of them were shown to engraft into the epithelial tubular area on days 3 and 14 after IR procedure. These findings suggest that angiogenesis occurred in peri-tubular region for possible tubular repair and regeneration after ADMSC transplantation

### Changes in mRNA Expression of Vasoactive, Inflammatory, Anti-oxidative, and Apoptotic Mediators in Renal Parenchyma after IR Injury

The mRNA expression of endothelin (ET)-1, an index of endothelial damage/vasoconstriction, was notably higher in group 2 than in groups 1 and 3, and significantly higher in group 3 than in group 1 (Table 1). These findings indicate that IR-induced renal endothelial damage was significantly suppressed through ADMSC treatment.

**Table 1:** Relative changes in mRNA expression of vasoactive, inflammatory, anti-oxidative, and apoptotic mediators in renal parenchyma after IR injury

Variables	Group 1 (n = 8)	Group 2 (n = 8)	Group 3 (n = 8)	p-value
Endothelin-1	1.00*	2.38 ± 0.48†	1.81 ± 0.34‡	<0.02
Tumor necrosis factor-α	1.00*	2.29 ± 0.25†	1.80 ± 0.20‡	<0.004
Matrix metalloproteinase-9	1.00*	1.48 ± 0.14†	1.19 ± 0.09‡	<0.05
endothelial nitric oxide synthase	1.00*	0.62 ± 0.19†	0.89 ± 0.16‡	<0.05
Interleukin-10	1.00*	2.06 ± 0.37†	2.86 ± 0.50‡	<0.03
Adiponectin	1.00*	0.50 ± 0.13†	0.75 ± 0.13‡	<0.004
NQO1	1.00*	1.89 ± 0.62†	2.76 ± 0.88‡	<0.04
Glutathione reductase	1.00*	1.67 ± 0.25†	2.54 ± 0.64‡	<0.04
Glutathione peroxidase	1.00*	1.83 ± 0.39†	2.82 ± 0.98‡	<0.04
Bcl-2	1.00*	0.74 ± 0.09†	0.94 ± 0.10‡	<0.02
Caspase 3	1.00*	2.01 ± 0.20†	1.53 ± 0.14‡	<0.05

Data are expressed as mean ± SD.

NQO = NAD(P)H quinone oxidoreductase

Group 1 = Normal control; Group 2 = Ischemia reperfusion; Group 3 = Ischemia reperfusion + adipose-derived mesenchymal stem cells

Symbols (*, †, ‡) indicate significant difference (at 0.05 level) by Bonferroni multiple-comparison post hoc test.

The mRNA expressions of tumor necrosis factor (TNF)-α and matrix metalloproteinase (MMP)-9, two indicators of inflammation, were remarkably higher in group 2 than in groups 1 and 3, and notably higher in group 3 than in group 1 (Table 1). On the other hand, the mRNA expressions of endothelial nitric oxide synthase (eNOS), IL-10, adiponectin, the anti-inflammatory indexes, were notably lower in group 2 than in group 3 (Table 1). These findings imply that ADMSC therapy inhibited inflammatory reaction in this experimental setting.

The mRNA expressions of NQO1, GR, and GPx, three anti-oxidative indicators, were remarkably lower in group 1 than in groups 2 and 3, and notably lower in group 2 than in group 3 (Table 1). These findings suggest an anti-oxidative response after induction of IR injury and an enhancement of anti-oxidant effect following ADMSC administration.

The mRNA expression of caspase 3, an index of apoptosis, was notably higher in group 2 than in groups 1 and 3, and markedly higher in group 3 than in group 1 (Table 1). In contrast, the mRNA expression of Bcl-2, an index of anti-apoptosis, was remarkably lower in group 2 than in groups 1 and 3, and significantly lower in group 3 than in group 1 (Table 1). These findings imply that ADMSC treatment exerted anti-apoptotic effects.

### Protein Expressions of Inflammatory and Anti-oxidative Mediators in Renal Parenchyma after IR Injury

**Figure 6:**
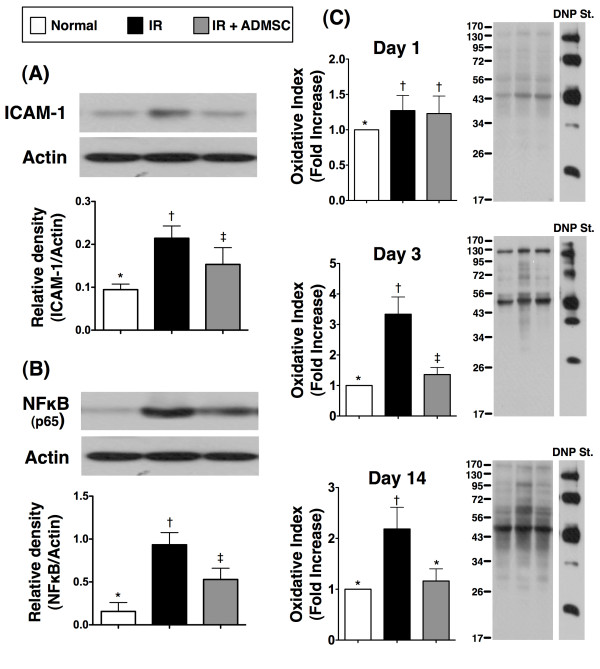
**Changes in protein expressions of inflammatory and oxidative markers in kidney after ischemia-reperfusion (IR)**. **A) **Remarkably higher expression of intercellular adhesion molecule (ICAM)-1 in IR group than in control and IR + ADMSC group, and notably higher in IR + ADMSC group than in control group; *p < 0.05 between indicated groups. **B) **Significantly higher expression of nuclear factor (NF)-κB in IR group than in control and IR + ADMSC group, and notably higher in IR + ADMSC group than in control group; *p < 0.04 between indicated groups. **C) **On day 1 after IR injury, lowest oxidative index, protein carbonyls, in the normal control group without significant difference between IR group and IR + ADMSC group; By day 3 after IR injury, notable increase in oxidative index in IR group compared with control group and IR + ADMSC group. Marked elevation also noted in IR + ADMSC group compared with control group; By day 14 after IR injury, oxidative index remarkably higher in IR group than in control and IR + ADMSC groups without significant difference between IR + ADMSC and control groups. **1) **Normal vs. day 1, *p < 0.05 between indicated groups; **2) **normal vs. day 3, *p < 0.05 between indicated groups; **3) **normal vs. day 14, *p < 0.02 between indicated groups. Symbols (*, †, ‡) indicate significance (at 0.05 level) (by Bonferroni multiple comparison post hoc test) (n = 8 for each group).

Western blot analyses (Figure 6) demonstrated remarkably higher protein expressions of ICAM-1 (A) and NF-κB (B), two inflammatory biomarkers, in group 2 than in groups 1 and 3, and in group 3 compared with those in group 1 at 72 h following acute renal IR injury. The protein expression of oxidative stress (Figure 6C), an indicator of ROS activity, did not differ between groups 2 and 3. However, it was remarkably higher in groups 2 and 3 than in group 1 at 24 h after IR injury. Additionally, it was increased several folds in group 2 as compared with that in groups 1 and 3 at 72 h and day 14 after IR procedure. Furthermore, it was notably higher in group 3 than in group 1 at 72 h but no difference was noted between groups 1 and 3 at day 14 after IR injury.

**Figure 7:**
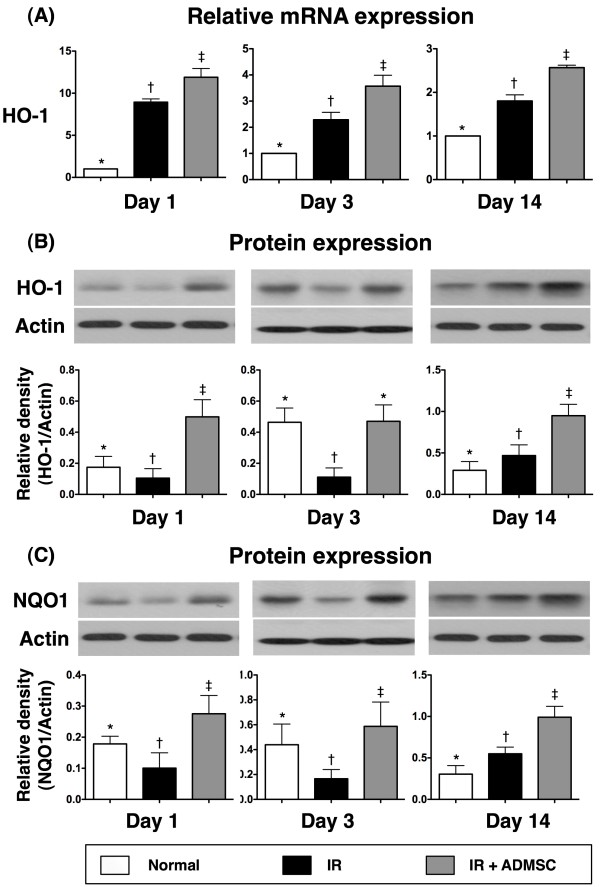
**The mRNA and protein expressions of anti-oxidative markers**. **A) **By days 1, 3 and 14 after IR injury, remarkably higher mRNA expression of heme oxygenase (HO)-1 expression in IR and IR + ADMSC groups than in control group, and notably higher expression in IR + ADMSC group than in IR group. **1) **Normal vs. day 1, *p < 0.0001 between indicated groups; **2) **normal vs. day 3, *p < 0.001 between indicated groups; **3) **normal vs. day 14, *p < 0.001 between indicated groups. **B) **By days 1 and 3 after IR injury, notably higher HO-1 protein expression in control and IR + ADMSC groups than in IR group, and significantly higher expression in IR + ADMSC group than in control group on day 1 after IR injury. Similar expressions, however, noted between control and IR + ADMSC groups by day 3 after IR procedure. By day 14 after IR, substantially higher HO-1 protein expression in IR + ADMSC group than in IR and control groups, and notably higher expression in IR group than in control group. **1) **Normal vs. day 1, *p < 0.01 between indicated groups; **2) **normal vs. day 3, *p < 0.01 between indicated groups; **3) **normal vs. day 14, *p < 0.01 between indicated groups. **C) **By days 1 and 3 after IR procedure, notably higher NAD(P)H quinone oxidoreductase 1 (NQO1) expression in control and IR + ADMSC groups than in IR group, and significantly higher expression in IR + ADMSC group than in control group; By day 14 after IR injury, notably higher NQO1 protein expression in IR + ADMSC and IR groups than in control group, and expression also significantly higher in IR + ADMSC group than in IR group. 1) Normal vs. day 1, *p < 0.03 between indicated groups; 2) normal vs. day 3, *p < 0.05 between indicated groups; 3) normal vs. day 14, *p < 0.01 between indicated groups. Symbols (*, †, ‡) indicate significance (at 0.05 level) (by Bonferroni multiple comparison post hoc test) (n = 8 for each group).

The mRNA expression of HO-1 (Figure 7A), an anti-oxidative biomarker, was remarkably higher in group 3 than in groups 1 and 2, and notably higher in group 2 than in group 1 at 24 h, 72 h, and day 14 after IR injury. Additionally, the protein expression of HO-1 (Figure 7B) was substantially lower in group 2 than in groups 1 and 3, and notably lower in group 1 than in group 3 at 24 h after IR injury. Moreover, the protein expression was remarkably lower in group 2 than in groups 1 and 3, but it did not differ between groups 1 and 3 at 72 h after IR injury. In contrast, it was notably lower in group 1 than in groups 2 and 3, and significantly lower in group 2 than in group 3 on day 14 after IR procedure.

The protein expression of NQO1 (Figure 7C), another anti-oxidative biomarker, was remarkably lower in group 2 than in groups 1 and 3, and significantly lower in group 1 than in group 3 at 24 h and 72 h after IR injury. On the other hand, this protein expression was markedly lower in group 1 than in groups 2 and 3, and notably lower in group 2 than in group 3 at day 14 after IR injury.

**Figure 8:**
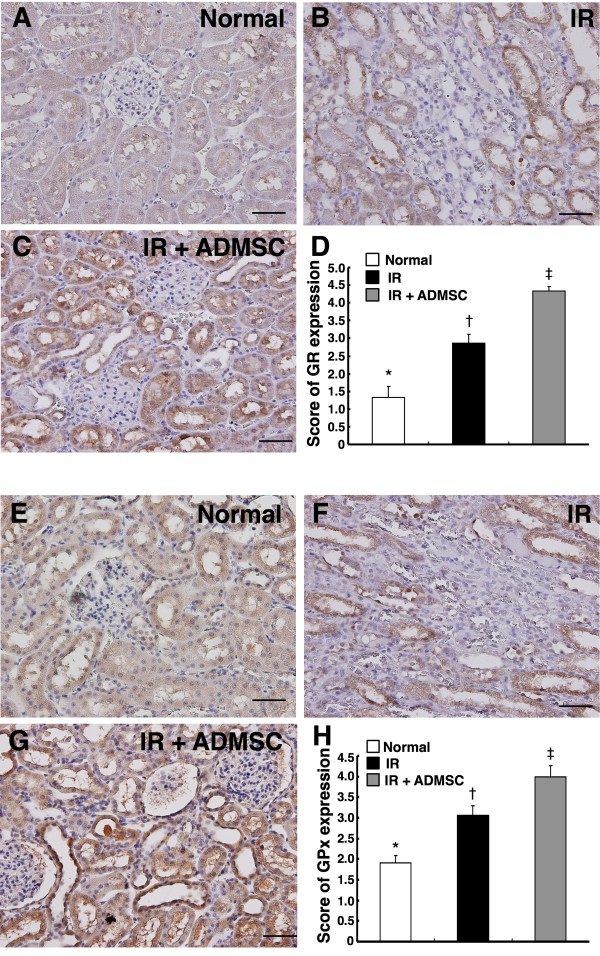
**Immunohistochemical staining for renal expressions of anti-oxidative markers**. Significantly lower score of glutathione reductase (GR)-positive cells (brown) in **(A) **Control group, than in **(B) **IR group, and **(C) **IR + ADMSC group. **(D) **Remarkably lower GR expression in IR group than in IR + ADMSC group. *p < 0.01 between indicated groups **(Upper panel)**. Significantly lower score of glutathione peroxidase (GPx)-positive cells (brown) in **(E) **Control group, than in **(F) **IR group, and **(G) **IR + ADMSC group. **(H) **Remarkably lower GPx expression in IR group than in IR + ADMSC group. *p < 0.02 between indicated groups **(Lower panel)**. (n = 8 for each group) Scale bars at right lower corner represent 50 μm (200 ×).

Besides, IHC staining (Figure 8) revealed that the expressions of GR and GPx, two anti-oxidative enzymes, were remarkably higher in group 3 than in groups 1 and 2, and notably higher in group 2 than in group 1. These findings further suggest that anti-oxidative responses were elicited by IR injury and ADMSC treatment contributed to further anti-inflammatory and anti-oxidative effects after IR-induced renal injury in this study.

### Findings from Immunofluorescent and IHC Staining

**Figure 9:**
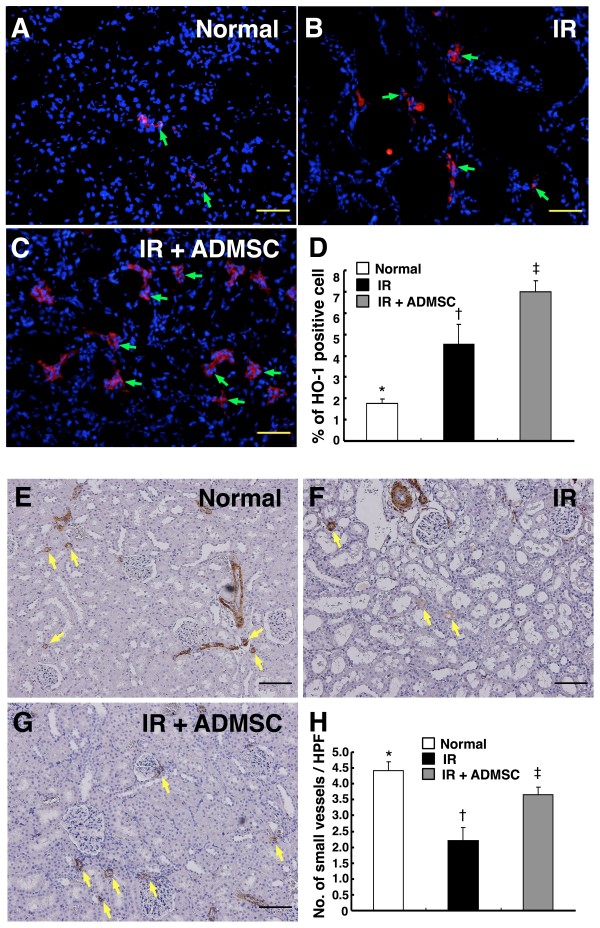
**Immunofluorescent staining for renal heme oxygenase (HO)-1 and alpha-smooth muscle actin (α-SMA) expressions after ischemia-reperfusion (IR)**. Remarkably reduced number of HO-1-positive cells (green arrows) in interstitial and peri-tubular area of kidney in **(A) **Control group, than in **(B) **IR group, and **(C) **IR + ADMSC group. **(D) **Notably lower number of HO-positive cells in IR group than in IR + ADMSC group. *p < 0.02 between indicated groups. Scale bars in right lower corners represent 50 μm (200 ×) **(Upper panel)**. Alpha-SMA staining showing notably higher number of small vessel (defined as diameter <25 μm) (yellow arrows) in **(E) **Normal controls, than in **(F) **IR group, and **(G) **IR + ADMSC group. **(H) **Significantly higher number of α-SMA-positive cells in IR + ADMSC group than in IR group. *p < 0.02 between indicated groups. Scale bars at right lower corner represent 100 μm (100 ×) **(Lower panel)**. (n = 8 for each group)

Immunofluorescent staining revealed remarkably higher number of HO-1-positive cells, an indicator of anti-oxidative status, in interstitial and peri-tubular area of kidney in group 3 than in groups 1 and 2 (Figure 9). On the other hand, the number was notably higher in group 2 than in group 1. Moreover, staining for α-smooth muscle actin showed that the number of small vessel was notably higher in group 1 than in groups 2 and 3, and significantly higher in group 3 than in group 2. These findings further suggest enhancement of anti-oxidant and angiogenesis effects as well as protection of small vessel from IR injury through ADMSC treatment.

## Discussion

This study, which used a rat model to investigate the therapeutic impact of ADMSC therapy on IR-induced acute kidney injury, provided several striking implications. First, ADMSC treatment significantly preserved architectural integrity of renal parenchyma and attenuated the deterioration of renal function after IR injury. Second, ADMSC therapy initiated early-onset anti-inflammatory and anti-oxidative effects. Third, the transplanted ADMSCs participated in angiogenesis early after IR injury.

### Stem Cell Therapy Effectively Protected Renal Function from Acute Kidney Injury--Complete Mechanisms Remain Poorly Defined

Surprisingly, while studies on animal models [6,7,10,11,13] have emphasized stem cell therapy as an effective treatment modality for acute kidney injury, the mechanistic basis underlying the observed improvement in renal function after stem cell therapy has not been extensively explored. In fact, the majority of previous studies [6,7,10-15] have focused on investigating one particular mechanism rather than a complete picture. The mechanisms having been established by previous studies included angiogenesis, stem cell homing, anti-inflammatory reaction, anti-oxidative stress, and immunomodulation [6,7,10-15,23]. The actual mechanisms underlying the improvement of renal function following ADMSC therapy could be even more complex [24]. The significance of a single mechanism within the whole picture, however, is still unclear [24].

### ADMSC Transplantation Attenuates Inflammatory Response, Suppresses Oxidative Stress, and Limits Cellular Apoptosis and Architectural Damage in Kidney Following Acute IR Injury--Impact of Immune Modulation

Numerous studies [6,7,24] have shown that acute IR injury elicits rigorous inflammatory response and enhances generation of ROS and free radicals which, in turn, cause the damage of renal parenchyma and destroy the architectural integrity of the kidney. One important finding in the current study is that the mRNA expressions of TNF-1α and MMP-9 as well as the protein expressions of ICAM-1, NF-κB, and oxidative stress were remarkably increased in group 2 compared to normal controls after acute IR injury. In this way, our findings reinforce those of previous studies [6,7,24]. Of importance is that, as compared with IR-injured animals without treatment, the expressions of these inflammatory and oxidative biomarkers at both gene and protein levels were significantly suppressed in animals following ADMSC administration. Accumulating evidence has demonstrated that MSCs have distinctive immunomodulatory property that contributes to the down-regulation of inflammatory reaction in ischemic condition [16,20,25]. Therefore, our findings are consistent with those of previous studies [16,20,25].

There are several key findings in the current study. Immunofluorescent and IHC staining as well as Western blot analysis demonstrated remarkably suppressed expressions of NQO1 and HO-1, the scavengers for free radicals, in group 2 and significant restoration in group 3 after ADMSC treatment. Besides, significant reduction was noted in the expressions of anti-oxidative enzymes GR and GPx in group 2 after IR injury, whereas the expressions were notably enhanced in group 3 following ADMSC therapy. In addition, significantly reduced mRNA expression of caspase-3 and notably enhanced mRNA expressions of Bcl-2 were demonstrated in IR-injured animals with ADMSC treatment compared with those without. Importantly, histological and serum biochemical findings showed, respectively, that renal parenchymal damage and renal dysfunction were substantially improved in group 3 following ADMSC treatment. Taken together, these findings suggest that ADMSC treatment preserved renal function, at least in part, through inhibiting inflammatory reactions, reducing apoptosis, and suppressing oxidative stress in this experimental setting of acute kidney IR injury.

The cause of discrepancy between mRNA and protein expressions of HO-1 on both day 1 and day 3 after IR remains unknown. We propose that it may be due to post-transcriptional regulation (i.e. speed of translation or degradation of HO-1) on mRNA and protein expression levels or the change in speed of protein degradation. Previous study by Mahin et al. [26] showed a synchronized up-regulation of HO-1 mRNA and protein levels at acute phase after IR. Thus, our results on mRNA expression rather than protein expression of HO-1 were consistent with their findings. Another possible explanation may be that the microsomal fraction was utilized for Western blot analysis in the study by Mahin et al. [26], whereas whole cell lysates were used for Western blot in our study. Since alternation in sub-cellular distribution of HO-1 has been reported in cell under stress [27], we speculate that the increased protein expression of HO-1 in the microsomal fraction of kidneys following IR injury [26] may be due to translocation of HO-1.

### Enhanced Angiogenesis through ADMSC Transplantation--An Ischemia-Relieving Phenomenon Accounting for Tubular Regeneration

**Figure 10:**
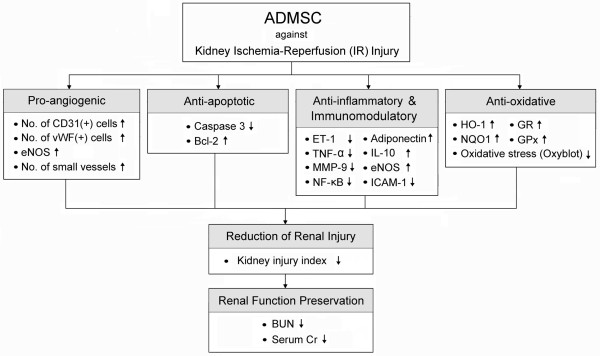
**Proposed mechanisms underlying the positive therapeutic effects of adipose-derived mesenchymal stem cell (ADMSC) on kidney ischemia-reperfusion (IR) injury**. ET-1: Endothelin-1; TNF-α: Tumor necrosis factor-α; MMP-9: Matrix metalloproteinase-9; NF-κB: Nuclear factor kappa B; IL-10: Interleukin-10; eNOS: Endothelial nitric oxide synthase; ICAM-1: Intercellular Adhesion Molecule-1; HO-1; Heme oxygenase-1; NQO1: NAD(P)H quinone oxidoreductase; GR: Glutathione reductase; GPx: Glutathione peroxidase.

Previous studies have demonstrated angiogenesis/vasculogenesis as one of the essential mechanisms underlying the improvement of ischemic organ dysfunction after stem cell therapy [12,13,16,20,28]. In the present study, we found that both CM-Dil-positive cells and cells positive for endothelial markers (i.e. CD31 and vWF), were abundantly present in interstitial and peri-tubular areas in animals having receiving ADMSC treatment. Furthermore, peri-tubular microvasculature was frequently observed in ADMSC-treated animals at 72 hour and day 14 after IR injury. Moreover, eNOS mRNA expression, an indicator of angiogenesis, was remarkably increased in group 3 animals after ADMSC administration. Consistent with our findings, a recent study using an immunodeficient mouse model of renal IR injury [7] has revealed that systemic administration of mobilized human CD34+ cells from peripheral blood reduced mortality, and promoted rapid renal repair and regeneration through paracrine effects on peri-tubular capillaries. Taken together, our findings, in addition to corroborating those of previous [12,13,16,20,28,29] studies, suggest that ADMSC treatment may improve renal function after IR injury through enhanced angiogenesis [28]. In summary, the possible mechanisms of ADMSC therapy in protecting kidney from IR injury are schematically presented in Figure 10.

### Study Limitations

This study has limitations. First, since the present study focused on the therapeutic impact of ADMSC on acute renal injury using a rodent model of renal IR, the effects of ADMSC administration were followed only up to 14 days after IR injury without looking into the chronic influence of treatment. Second, although our findings are promising, the underlying mechanisms (Figure 10) involved in ADMSC therapy against renal IR injury remains descriptive. Further investigations, therefore, are warranted to clarify the exact mechanisms involved.

## Conclusions

ADMSC markedly improved renal function after acute IR injury. The key mechanisms underlying the positive therapeutic impact of ADMSC treatment on renal function could be due to suppression of inflammatory response and oxidative stress as well as enhancement of angiogenesis.

## Competing interests

The authors declare that they have no competing interests.

## Authors' contributions

All authors have read and approved the final manuscript.

YTC, YHK, YCL, BCC, and CKS designed the experiment, performed animal experiments, and drafted the manuscript. LTC, THT, YLC, SC, PLS, and CHY were responsible for the laboratory assay and troubleshooting. KCC, SL, and HKY participated in refinement of experiment protocol and coordination and helped in drafting the manuscript. Yu-Chun Lin contributed equally as the first author to this work. Steve Leu contributed equally compared with the corresponding author to this work
